# Community engagement and involvement in Sri Lanka: a knowledge mobilisation case study

**DOI:** 10.1186/s40900-026-00923-3

**Published:** 2026-06-16

**Authors:** Manouri Wimalasekera, Steven Blackburn, Risitha Wakishta Arachchi, Chamali Jayasinghe, G. N. Duminda Guruge, Adele Higginbottom, Oshini Sri Jayasinghe, Sameeha Jabir, Vidath Samarakkody, Janani Opatha, Lasith Dissanayake, Kaushalya Jayaweera, Enoka Wickramasinghe, Asiri Hewamalage, Buddhika Fernando, Sunil de Alwis, Godwin Kodituwakku, Nihal Abeysinghe, Helena Zavos, Krysia Dziedzic, Athula Sumathipala

**Affiliations:** 1 Institute for Research & Development in Health and Social Care, Colombo, Sri Lanka; 2 University of Birmingham, Birmingham, UK; 3 Department of Health Promotion, Rajarata University of Sri Lanka, Mihintale, Sri Lanka; 4 Keele University, Newcastle-under-Lyme, UK; 5 Health Promotion Bureau, Ministry of Health, Colombo, Sri Lanka; 6 Family Health Bureau, Colombo, Sri Lanka; 7 Education, Training & Research Unit, Ministry of Health, Colombo, Sri Lanka; 8 Colombo, Sri Lanka; 9 Institute of Psychiatry, Psychology & Neuroscience, King’s College, London, UK

**Keywords:** Engagement, Involvement, Community, Research, Public, Health, Ethics, Collaboration, Stakeholders, PPI, PPIE, CEI, Sri Lanka

## Abstract

This paper provides a narrative case study of how Community Involvement and Engagement was introduced and embedded within the Sri Lanka research system between 2018 and 2025. Using stakeholder engagement activities, workshop outputs, and programme documentation coordinated by the Institute for Research and Development in Health and Social Care (IRD), the study explores the methodologies, processes, and institutional strategies that supported sustained CEI development in a lower middle-income country (LMIC). The paper demonstrates that successful CEI requires addressing structural inequalities and fostering deep community engagement. Embedding CEI requires alignment between ethical governance, institutional leadership, and culturally adapted engagement practices, underpinned by knowledge mobilisation. The findings highlight the potential for bidirectional benefits between lower- and higher-income countries, which may contribute to evolving and improving the the research culture in LMICs. These efforts have laid a robust foundation for CEI in Sri Lanka, serving as a model for other LMICs. Sustaining this momentum will require continued innovation, strategic partnerships, and integration of CEI into national research policies.

**Supplementary Information:**

The online version contains supplementary material available at 10.1186/s40900-026-00923-3.

## Abstract (plain language summary)

This paper describes how researchers in Sri Lanka have worked with communities to shape health and social care research. This approach, called Community Engagement and Involvement (CEI), makes sure that research projects and programmes work in partnership with the public, helping to make research more relevant, meaningful, and useful to the people and communities intended to benefit from it. The Institute for Research and Development (IRD) in Colombo, Sri Lanka led several activities to promote CEI across research institutions, and professional and public communities throughout Sri Lanka. These included workshops, public events, and the creation of community groups where local people could share their views and help guide research decisions. The IRD also worked with local and international partners to share knowledge and resources, which strengthened the impact of their efforts. One important achievement was bringing CEI into the ethical review of research projects. This means that community perspectives were considered right from the start of planning new studies. Although political and economic challenges made the work difficult, the IRD showed that CEI can improve both the quality and usefulness of research. Their experience provides lessons for other countries aiming to build stronger partnerships between researchers and communities. The IRD made an impact by promoting the concept among wider community in Sri Lanka.

**Supplementary Information:**

The online version contains supplementary material available at 10.1186/s40900-026-00923-3.

## Background

The IRD in Sri Lanka has been engaged in community-focused initiatives since its inception in 1997. However, formalised, Community Involvement and Engagement (CEI) structures, institutional frameworks and practices were largely absent prior to 2018, although community-oriented approaches existed within public health and social research undertaken by the IRD. The introduction and institutionalisation of CEI have therefore occurred recently, primarily driven by the Institute for Research and Development in Health and Social Care (IRD) and its collaborators, particularly Keele University, UK.

### Definition of CEI

Working with the community throughout the research cycle; from the inception to develop conduct and disseminate research, ensures that it is relevant to and shaped by the people and community that the research is focused on [1]. In this paper, Community Engagement and Involvement (CEI), is used as the overarching term to describe participatory approaches involving and engaging with communities throughout the research process. The terms Patient and Public Involvement and Engagement (PPIE) and Public Engagement (PE) are retained where historically accurate, particularly when referring to workshops, funding terminology, or frameworks that originally used those terms. These terms are sometimes used interchangeably [2] in global health research. CEI is defined as the active involvement of communities throughout the research process, using participatory approaches and working in partnership with key stakeholders to improve the relevance, value, and conduct of health research [3]. In the Sri Lankan context, this terminology is more inclusive, extending beyond “patients” to encompass families, caregivers, and wider community groups.

### Theoretical underpinnings

This study is informed by participatory, co-production, and epistemic justice theories, which emphasise redistribution of power, recognition of lived experience, and shared decision-making between researchers and communities [4]. Within this framework, CEI is conceptualised as a relational and collaborative process through which diverse forms of knowledge enables communities to actively influence research agendas, design, and implementation.

Evidence demonstrates that the quality and appropriateness of research is enhanced when it incorporates CEI, and that implementation of findings is improved when the public are actively engaged [5]. Globally, CEI is increasingly seen as a fundamental element of research because it places communities and public members at the centre of outcomes, ensuring that scope, processes, and evaluation are relevant and beneficial to end-users. Importantly, the public frequently prioritises research topics differently from academics and professionals [6], making CEI critical for shaping research agendas that are relevant for the needs of people and communities.

Our approach draws on knowledge mobilisation principles [7, 8] to ensure that findings are co-produced and implemented with communities, as well as co-production frameworks to build equitable partnerships. We also align with participatory health research traditions, which emphasise shared decision-making, co-production and collective ownership of research [9].

### Concepts and theory development

Keele University has been a leader in PPIE in the UK since 2006, developing Community Research Involvement Groups (CRIGs) that embed lived experience across the research lifecycle [10]. With over 180 public contributors, this model demonstrates meaningful public involvement research design, delivery, and dissemination.

Core CRIG principles, including co-production and race equality [11], informed the adaptation of CEI in Sri Lanka. This illustrates how frameworks from high-income settings can be translated into culturally appropriate practices in LMIC contexts.

### Global impact of CEI

The presence and role of CEI in research varies globally [12]. It is well established in the United Kingdom, Netherlands, United States, Canada, and Australia, where national organisations such as the National Institute of Health and Social Care Research (NIHR) (UK), Patient-Centred Outcomes Research Institute (USA), and Strategy for Patient-Oriented Research (Canada) provide infrastructure for CEI. Elsewhere, CEI is emerging or absent, often hindered by limited resources, institutional support, or understanding [13]. To strengthen practice, initiatives such as the NIHR have introduced the International Patient and Public Involvement Network and Guiding Principles for CEI in global health research [14].

### Institute for research and development in health and social care research

The Institute for Research and Development in Health and Social Care Research (IRD), established in 1997, is a not-for-profit, independent research institution in Sri Lanka. Its academic members span multiple disciplines, including epidemiology, psychiatry, medicine, genetics, veterinary science, public health, ethics, education, and social sciences. With full-time staff and an extensive network of national and international collaborators, IRD’s mandate is to foster a strong research culture by conducting multidisciplinary studies, building strategic partnerships, and working for policy impact. Since its inception, IRD has maintained a “with the people” approach, exemplified by its pioneering twin studies and the creation of the first population-based twin registry in LMICs, now including over 10,000 pairs. In recognition of its collaborative ethos, IRD received Sri Lanka’s National Award for Science and Technology in 2019 for Excellence in International Collaboration.

CEI in Sri Lanka has undergone a transformative journey between 2018 to 2025(Table 1), with IRD playing a central facilitating role in institutionalising involvement through sustained local leadership, international collaboration, stakeholder and community engagement, and system-level knowledge mobilisation. Earlier participatory and public engagement activities conducted prior to 2018 are presented as foundational precursors that informed the later development of structured CEI approaches in Sri Lanka (Table 2).

**Table 1:** IRD CEI/PPIE milestones overview from 2018 to 2025

July 25, 2018	SLMA 131st annual scientific sessions pre-congress PPIE workshop.
August 24, 2018	PPIE small group meeting at IRD office.
October 13, 2018	Cultural Centre (Ape Gama) twin public gathering.
February 1, 2019	Twin school children gathering at Defence Services School, Colombo.
February 5, 2019	Ethics committee members of Universities/Ministry of Health - PPIE workshop.
February 6, 2019	Follow-up PPIE workshop for participants at 131st SLMA (Sri Lanka Medical Association).
February 7–8, 2019	PPIE Workshop at Department of Health Promotion, Rajarata University of Sri Lanka.
July 22, 2019	Sri Lanka Medical Association (SLMA) 132nd annual scientific sessions pre-congress PPIE workshop.
December 14, 2019	Colombo Twin and Singleton Study 3 (COTASS 3) Twin lay member CEI CRIG meeting at IRD office.
March 21, 2021	Introducing Public involvement concept to COTASS 3 research team virtual Workshop via Zoom.
July 2, 2021	Open University of Sri Lanka (OUSL) PPIE virtual workshop via Zoom.
August 28, 2021	Kotalawala Defence University (KDU) PPIE workshop via Zoom.
January 31, 2022	Enhance project PPIE workshop for Midwives at the office of Medical Officer of Health, Battaramulla.
March 3, 2022	COTASS 3 project - workshop for lay group training at Rajarata University of Sri Lanka.
August 17, 2022	Virtual workshop for Ethics Review Committee of Institute of Indigenous Medicine, University of Colombo (ERCIIM of UOC) via Zoom.
November 22, 2022	Colombo Medical College, University of Colombo onsite PPIE / CEI workshop (Colombo Medical Congress 2022 pre-congress workshop).
December 16, 2022	CEI/PPIE Webinar for Research Council of Gampaha Wickramarachchi University of Indigenous Medicine (GWUIM).
January 18, 2023	CEI introductory webinar for the Department of Social Sciences and Humanities, Rajarata University of Sri Lanka.
February 11, 2023	Twin Focus Group Discussion about the Social Media Campaign on the Sri Lanka Twin Registry (SLTR) at the Water’s Edge, Battaramulla.
November 11, 2023	University of Peradeniya Postgraduate Institute of Agriculture (PGIA) onsite CEI hybrid workshop.
January 6, 2025	Focus Group Discussions were held to finalize the plot and direction of a potential drama about perinatal depression. Participants included mothers with lived experience, family members, and other stakeholders.
March 7, 2025 April 17, 2025	A docudrama on Perinatal Depression was made as part of the ENHANCE project and screened for the first time. The docudrama was uploaded on YouTube
June 14, 2025	Twin Talent Fest 2025, a talent show for twins, was held as a lead-up to the International Society for Twin Studies Congress 2025(ISTS 2025)
July 31 2025	TRANSLATE Stakeholder Engagement and Consensus Building Workshop
August 10–12, 2025	10 Mentees were chosen and offered free participation for the ISTS 2025, as part of CEI and Capacity Building efforts.
October 2, 2025	The participants of the Twin Talent Fest 2025 were invited for another engagement session at the IRD

**Table 2:** Pre-CEI foundations and participatory precursors

Year	Event
December 2009	Multiples Engaging in Research through Culture Continuity (MERC) - First stage of the cultural festival among twins, competitions in dancing, singing, drawing, essay writing, etc. under the theme “art for scientific research”.
2010	IRD received a public engagement grant from the Wellcome Trust for the twin research in collaboration with Kings College.
2010	Multiples Engaging in Research through Culture Continuity (MERC) - Second stage of the cultural festival among twins.
2012	A workshop on singing traditional musical instruments and cultural dancing was held for twins participating in the MERC competitions.

Sri Lanka has begun to develop its own CEI structures, with the IRD playing a pioneering role. This paper presents a narrative of how CEI has been introduced and embedded in Sri Lanka through sustained knowledge mobilisation at national, societal and practitioner levels, highlighting the methodologies, processes, and practices that supported its development. By documenting these experiences, the paper provides evidence-based insights into sustaining meaningful CEI in a lower-middle-income country and contributes to the wider global discussion on adapting involvement frameworks to diverse contexts.

## Methods

### Study design

This study adopts a narrative case study design to examine how Community Engagement and Involvement (CEI) was introduced and embedded within the Sri Lankan research ecosystem between 2018 and 2025. Narrative case studies are well suited to examining complex system changes that are underpinned by institutional, cultural, and relational processes [15].

The approach is informed by knowledge mobilisation principles and aims to generate practice-based insights into embedding CEI within a lower-middle-income country context.

### Aim and objectives

The overall aim of this initiative was to establish and embed CEI practices within the Sri Lankan research context.

The specific objectives were to:

1. Initiate awareness raising and shared understanding of CEI in research.
2. Recruit a critical mass of engaged professionals and informed public members.
3. Establish coordination mechanisms within IRD to sustain CEI activity.
4. Develop culturally adapted training materials and communication to support the involvement of community members as research partners.
5. Extend CEI practice and expertise of CEI through national and international collaborations and shared learning.

### Scope, data sources & stakeholders

The case study focuses on CEI activity coordinated or supported by the Institute for Research and Development in Health and Social Care (IRD) at national, institutional, practitioner, and community levels across Sri Lanka. Data sources comprised IRD CEI programme records; workshop reports and training outputs; project documentation from IRD‑led and supported studies; and institutional reflections arising from sustained stakeholder engagement. A wide and diverse group of Sri Lankan stakeholders participated in CEI-focussed activities facilitated by IRD, including:

- Community members: study participants, CRIG members and community groups associated with several IRD research projects covering twin health, nutrition, and mental health topics [16–18].
- Academics, researchers and students.
- Ethics Review Committee members.
- Health professionals.
- Journalists and broadcasters.
- International collaborators: Keele University, King’s College London, the UK Health Research Authority, NIHR UK, and project partners from Brazil and Ethiopia for the ECLIPSE project [19].

Together, these groups contributed to priority-setting, study design, recruitment, ethical review, training, implementation, and dissemination.

### CEI implementation framework

CEI activities were analytically organised using a four-stage implementation framework, which supported synthesis across diverse initiatives:

1. Awareness-raising and agenda setting – introducing CEI concepts across academic, policy, and community settings through high-profile workshops, events, and media.
2. Design and planning – integrating CEI into study conception through Community Research Involvement Groups (CRIGs) and stakeholder collaboration, informing culturally appropriate study protocols and intervention design.
3. Implementation – involving community partners in tool development, recruitment approaches, and governance mechanisms.
4. Dissemination – using academic, community, cultural, and media channels to ensure accessible knowledge exchange.

This framework functioned as a flexible analytical lens, rather than a prescriptive linear model, as CEI activities overlapped across awareness-raising, design, implementation, and dissemination stages. Empirical examples illustrating each stage are presented in the Results and summarised in Table 3.

**Table 3:** Mapping of study objectives to CEI implementation framework stages and illustrative outputs

Objective	Main Framework Stage(s)	Examples
Awareness raising	Stage 1	SLMA workshops, twin gatherings
Recruit professionals/public	Stages 1–2	ERC workshops, CRIG formation
Coordination mechanisms	Stages 2–3	CEI small group, IRD CEI group
Training/adaptation	Stages 3–4	Trilingual materials, CRIG training
Extend expertise	Stages 1–4	ENHANCE, ECLIPSE collaborations

## Results

Results are organised according to the five stated objectives, with activities and outcomes mapped onto the four‑stage CEI implementation framework: awareness‑raising, design and planning, implementation, dissemination.(see Table 3) All examples presented are drawn from IRD-led or IRD-supported projects, unless otherwise stated.

### Objective 1: Initiate awareness raising and shared understanding of CEI in research (primarily implementation stage 1: awareness-raising and agenda setting)

#### Initial engagement with researchers and the academic community

Awareness-raising formed the foundation for CEI development in Sri Lanka. Between 2018 and 2023, IRD coordinated a series of national, institutional, and sector-specific activities aimed at introducing CEI concepts to researchers, professionals, and communities.

At national level, CEI was introduced through high‑profile academic platforms, beginning with a pre‑congress workshop at the 131st Annual Scientific Sessions of the Sri Lanka Medical Association (SLMA) in July 2018, followed by repeat sessions in 2019 and subsequent workshops at the Colombo Medical Congress in 2022. SLMA is the umbrella organisation of the medical professionals and academics in Sri Lanka. The workshop was held on 25th July 2018 and was titled, ‘Establishing and sustaining meaningful Patient and Public Involvement and Engagement (PPIE) in research’. It was jointly organised by SLMA, IRD, Rajarata University of Sri Lanka (RUSL) and Keele University UK. Two CEI experts from Keele University, UK contributed as key resource persons. To ensure a broad and diverse group of professionals attend the workshop, individuals were selected and invited from those who registered for the pre-congress workshops through the conference website. Seventy-two participants attended the workshop which included medical and health professionals, academics, human rights experts, media professionals, and informed members of the public.

A follow-up workshop was held in February 2019 at the National Institute of Mental Health (NIMH), Sri Lanka. Participants from the 2018 SLMA pre-congress workshop were invited, along with other interested professionals and academics who had inquired about the event. A total of twenty-three individuals attended this session, which provided an opportunity to reflect on their experiences and share knowledge approximately one year after their initial exposure to the concept of Community Engagement and Involvement (CEI). Participants discussed practical challenges they had encountered when applying CEI principles in their respective professional contexts. Three CEI experts, from the UK’s Health Research Authority (HRA) and Keele University, shared their insights during the workshop. Attendees included clinicians from the Sri Lankan state health sector, as well as academics from the field of indigenous medicine, among others.

There was sizable demand for CEI knowledge by 2019 and IRD was invited to organise a repeat pre-congress workshop at the 132nd annual scientific session of SLMA on 22nd July 2019. Two IRD affiliated Sri Lankan medical professionals who had the opportunity to train at the Keele University UK on CEI during their post-graduate studies contributed as the key resource persons at this workshop.

This was followed by a pre-congress workshop on CEI at Colombo Medical Congress in November 2022, which was organised by the Faculty of Medicine, University of Colombo (UOC) - the oldest and a well-recognised state institution for medical education in Sri Lanka. This workshop was opened not only to researchers and academics but also for students reading for undergraduate degrees pertinent to medicine. By this time IRD managed to source Sri Lankan researchers who have adapted CEI into their research to share their experiences at this workshop. These researchers adapted CEI into their work on community health promotion and an intervention program to improve patient journey and to reduce stigma via community education [19].

From the inception, IRD invited professionals working in the media industry to participate and cover CEI related events. National newspapers, electronic media and digital media were engaged in the journey as key stakeholders. The media offered its space free of charge recognising CEI’s value, which increased the reach of the online events organised by IRD. That was crucial as physical events that IRD could organise became limited due to COVID 19 pandemic in 2021–2022 period. The IRD’s trilingual quarterly journal, Gaveshana (meaning explorer) was effectively used to disseminate information pertinent to CEI among professionals, academics, undergraduate and secondary school students. A special issue on CEI was published in Gaveshana January – March 2021 [20].

#### Initial engagement with communities

The first community engagement event on CEI was held in Sri Jayewardenepura, Kotte, the administrative capital of Sri Lanka on 13th October 2018 with over 125 twin pairs, their parents and siblings participating by invitation. Celebrities and leading professionals who are twins or having twin children also attended. Another twin gathering was organised in a primary school, Defence Services School in Colombo on 1st February 2019. These events were to initiate the specific CEI activity of the SEARCH project. These activities opened a dialogue with the twin community on CEI and how they could contribute as a community towards research pertinent to twins. These twin gatherings were interactive and culturally engaging, facilitating community participation. They received widespread media coverage, often framed as human-interest stories, which helped communicate CEI concepts to a broader audience.

### Objective 2: Build a critical mass of engaged professionals and informed public members (implementation stages 1 and 2: awareness-raising; design and planning)

IRD identified Ethics Review Committee (ERC) members of Sri Lankan universities, institutes operating under the ministry of health and professional bodies as a critical group that need to be sensitised on CEI. The first such meeting was held on 05th February 2019 which was attended by senior academics representing ERCs of State universities and other institutions. They valued the notion of CEI and were convinced and agreed to take the message of CEI to the respective universities and institutions. At the meeting it was proposed that ERC members of universities and research institutions should be sensitised to CEI.

IRD received an invitation from the ERC of the Faculty of Indigenous Medicine, University of Colombo (FIM, UOC) to organise a virtual workshop on CEI and research ethics. Through that workshop IRD sensitised the ERC members of FIM, UOC and more than one hundred participants attended the online workshop which was held on 17th August 2022.

IRD had the opportunity to introduce CEI to the Research Council of Gampaha Wickramaarachchi University of Indigenous Medicine (GWUIM). IRD conducted an online workshop for their research council members and other interested researchers which was held on 16th December 2022. It was well received by the researchers attached to GWUIM research council. They participated in an insightful discussion during the question-and-answer session. GWUIM opened the workshop for academics and postgraduate researchers, which numbered more than sixty.

Introducing CEI to researchers specialised in Social Sciences and Humanities was a key milestone in the CEI journey in Sri Lanka. That was achieved through a webinar for the Department of Social Sciences and Humanities, RUSL which was held on 18th January 2023. That two-hour webinar paved the way to raise awareness on CEI among social scientists and undergraduates reading for social sciences and humanities.

The agriculture sector has produced impactful research in Sri Lanka (Gunawardena, 2020). Among plethora of research institutes in the sector, Postgraduate Institute of Agriculture (PGIA), University of Peradeniya (UOP) is well established and the leader in postgraduate training in Sri Lanka. IRD introduced CEI to agriculture scientists by way of a hybrid workshop conducted from PGIA, UOP, emphasizing the value of engaging with communities and local farmers in agriculture research. This three-hour hybrid onsite workshop was held on 11th November 2023 included attendees comprising of PGIA students, academic staff, and researchers.

Engagement of informed public members occurred through recruitment into Community Research Involvement Groups (CRIGs) formed around specific studies. These groups provided an entry point for lay contributors to move beyond consultation and into collaborative involvement from design stages onward. The first CRIG, formed in 2019 for the COTASS-3 nutrition and health study, consisted of nine lay twin participants [21]. They influenced study protocols, including a culturally sensitive recommendation that only female research assistants collect bio samples from female participants. This advice was formally adopted by the research team, illustrating genuine co-production.

Further design-stage involvement occurred in the *ENHANCE Sri Lanka* project on perinatal depression, where mothers with and without lived experience of perinatal depression, public health midwives, and other health professionals influenced the project direction, study design, co-adapting a psychosocial intervention to the Sri Lankan context [22], and in agricultural research where postgraduate students and academics engaged in CEI workshops at the Postgraduate Institute of Agriculture in 2023.

### Objective 3: Establish coordination mechanisms within IRD to sustain CEI activity (implementation Stages 2 and 3: design and planning; implementation)

To ensure continuity beyond individual projects, IRD established internal coordination mechanisms to support CEI across its research portfolio. After introducing the concept of CEI initially in July 2018 to Sri Lanka, IRD organised a meeting with professionals and public who showed high enthusiasm at SLMA CEI workshop to work together with IRD and function as a core group, promoting CEI with a long-term objective. The meeting was held on 24th August 2018. The members of the group discussed broadly to set the specific objectives for future work and suitable work plan and mechanism to move forward. This group continue to be the ‘informal decision making’ body for CEI activities in IRD.

IRD incubated this group through a virtual network which is called *‘IRD CEI small group’* and it was active throughout the COVID-19 pandemic, and the turbulent period Sri Lanka underwent in 2022 due to economic and political challenges. That allowed CEI related activities to continue and utilise the window of opportunity where relevant stakeholders were comparatively relieved from their routine responsibilities and engaging them with CEI activities.

In August 2018, IRD appointed a CEI coordinator to support and facilitate CEI activities. The key role helped to explore and utilise the opportunities to disseminate knowledge and share experiences pertinent to CEI with Sri Lankan and foreign experts.

Within research projects, IRD coordinated the involvement of community members with research teams and institutions. For example, in the Colombo Twin and Singleton Study (COTASS-3) nutrition and health project, a nine-member CRIG comprising lay twin participants met with researchers during design and implementation stages, contributing to decisions that shaped study procedures. CEI members tested research tools, co-facilitated training, and shaped recruitment strategies. For example, CRIGs trialled questionnaires to ensure clarity and cultural appropriateness, while ethics review committee sensitisation workshops in 2019 and 2022 enabled CEI considerations to be embedded in formal review processes.

IRD lobbied to include CEI at relevant state sector activities from 2018. In May 2022, Ministry of Health, Government of Sri Lanka established its first CEI subcommittee of the National Steering Committee on Childhood Developmental Disabilities, with IRD invited to the subcommittee.

### Objective 4: Share knowledge through training and culturally adapting materials (implementation stage 4: dissemination)

To disseminate knowledge on CEI and ensure appropriate involvement from the Sri Lankan communities, communications about CEI should be carried out not only in English language but also in commonly used local languages, Sinhala, and Tamil in Sri Lanka. Therefore, a small CRIG, reflective of different Sri Lankan communities, was formed to plan trilingual communications. IRD sought advice from experts in communication and language to introduce appropriate terminology. Email discussions were initiated at various stages to support appropriate translations and adaptations, considering cultural values, norms, and pronunciation challenges. In developing local language CEI content, IRD’s own publication Gaveshana was used to establish words and phrases which paved the way for other publishers to follow. There were occasions that local language terms that were introduced at the early stages of CEI journey in Sri Lanka were found inappropriate or inadequate and they were improved, and in some cases replaced.

In 2019 IRD commenced a project aiming to develop research capacity on ‘nutrition and health’ in Sri Lanka which was funded through the MRC, UK. The research project was conducted in collaboration with King’s College London (KCL), UK, Keele University, UK, RUSL, University of Colombo, Sri Lanka (UOC) and National Institute of Fundamental Studies (NIFS), Kandy, Sri Lanka. This study was the third wave of the Colombo Twin and Singleton Study (COTASS 3) conducted with the Sri Lankan Twin Registry, established by IRD. The project comprised five key objectives, one of which was to implement CEI in a research setting. To achieve this, the IRD established a CRIG by inviting participant twins, forming a nine-member group - the first CRIG initiated by IRD. To empower its members, a dedicated workshop was held on 14th December 2019 at the IRD office. The CRIG members contributed to the research from its designing stage which improved the quality of the research. For an example, a CRIG member proposed to pay attention to the socio-cultural sensitivity of the country and to allocate only female research assistants when collecting bio samples from female participants of the research. This proposal which was validated by certain other participants at the workshop was agreed and adapted by the research team. On 21st March 2021 IRD organised a virtual workshop with the participation of researchers involved in the COTASS 3 nutrition and health research project. That paved the way for a discussion and identified challenges in introducing CEI through CRIGs. On 3rd March 2022, IRD conducted a workshop in collaboration with Department of Health Promotion (DHP), Rajarata University of Sri Lanka (RUSL), Mihintale to train selected eleven lay members of the community enabling them to join CRIGs on nutrition related future research. This joint workshop was conducted in the local language, Sinhala which offered the confidence to IRD that they are equipped to share CEI knowledge in local languages.

Before the introduction of the CEI lexicon in Sri Lanka, the Department of Health Promotion (DHP), Rajarata University of Sri Lanka (RUSL) had introduced an approach of working ‘with’ and ‘by’ the community to improve their own health: a ‘community based health promotion approach’, where the general public were empowered to initiate and execute interventions in their own communities (Herath et al. 2017, Guruge et al. 2018). The Community Based Health Promotion process is driven by the targeted community, as the ownership of the process is with the community. Facilitators from the DHP, RUSL were available to help improving capacities and skills of people. DHP, RUSL being a longstanding partner of the IRD joined hands early in the CEI journey with them. That allowed IRD to gain from the experiences of DHP, RUSL and adopt CEI in a manner that could be grasped by grass root community members. CEI experts from Health Research Authority UK and Keele University UK along with IRD team paid a visit the communities working with DHP, RUSL in February 2019. This two-day workshop was organized both at DHP, RUSL main campus at Mihintale in the North Central Province (220 km away from Sri Lanka’s capital, Colombo) and at Medawachchiya, a rural village approximately 22 km away from the main campus. Rural Sri Lankan community’s ability to contribute and participate in scientific research were evident and from RUSL’s work which was identified as a base to propel CEI utilising the enthusiasm and commitment of the community.

Academics and researchers of Department of Community and Family Medicine, Faculty of Medicine, University of Jaffna, in its publication titled ‘Community Engagement and Involvement in Research: A local Experiences,’ in 2020 developed Tamil language CEI tools in their research project on Atrial Fibrillation (AF) management. The three case studies within the project provide a strong presence of CEI [23].

Dissemination was integral and continuous. Media partners volunteered free coverage of CEI activities, ensuring wide national reach. Academic dissemination occurred through local and international conferences, including a poster presentation at Keele University’s Research User Group event (2019). IRD also leveraged trilingual publications and digital campaigns, broadening accessibility across Sinhala, Tamil, and English-speaking communities. Public-facing outputs included cultural events, talent shows, and, more recently, a docudrama on perinatal depression developed with stakeholder input (2025).

IRD used legacy media and social media (i.e. Facebook, Instagram, TikTok) to inform the research community and the public at large on CEI activities which was well received. This was especially during the COVID pandemic period the continuous CEI activities via virtual platforms warranted impactful new media communication. IRD received a seed fund titled ‘Piloting the use of Social Media as a method of engaging participants in an LMIC population’ from MRC, UK which allowed it to obtain the services of outside expertise to upskill its content creators who handle CEI communication. With that IRD developed and piloted cost-effective methods for engagement and dissemination of information specially for the twin research.

### Objective 5: Extend CEI practice and expertise through national and international collaborations and shared learning (all implementation stages: awareness to dissemination)

National and international collaborations played a critical role in consolidating CEI practice. Partnerships with institutions such as Keele University, King’s College London, and NIHR-funded programmes provided methodological support, credibility, and opportunities for reciprocal learning.

IRD supported and joined a four-year multinational global health research study funded by NIHR, UK, to lead a global health program investigating the social impact of neglected tropical disease, Cutaneous Leishmaniasis. The research project titled, Empowering people with Cutaneous Leishmaniasis: Intervention Program to improve patient journey and reduce Stigma via Community Education – ECLIPSE, team are working in four countries viz. Brazil, Ethiopia, Sri Lanka and UK. RUSL led the research in Sri Lanka and Honorary Director IRD joined the project as co-principal investigator [24].

The ECLIPSE team is strongly committed to involving community members in all the ECLIPSE activities in partner countries. It places CEI at the heart of ECLIPSE. On 8th December 2023, IRD organised a public presentation to showcase CEI in the ECLIPSE project conducted in Sri Lanka and shared the experience with global partners. The event was held at the National Science Foundation, Colombo, Sri Lanka.

Thinking Healthy Programme (THP) is a low-intensity task-shifted psychosocial intervention for perinatal depression endorsed by the World Health Organization as the first- line of treatment for Perinatal depression [25]. THP has been producing promising results and high community engagement and acceptance in other LMICs such as Pakistan. In 2021, IRD researchers planned to commence similar intervention within Sri Lanka to address the treatment gap for perinatal depression.

Scaling-up Care for Perinatal Depression through Technological Enhancements to the THP (*ENHANCE Sri Lanka*) is the Sri Lankan arm of the ENHANCE programme funded by NIHR, UK.

A strong CEI component is included in the *ENHANCE Sri Lanka* project where Public Health Midwives were empowered with CEI knowledge during a workshop held at the office of Medical Officer of Health, Battaramulla, Sri Lanka on 31st January 2022. Sixteen health care professionals attended this workshop where they contributed to deciding the project implementation and designing the research. The knowledge that is gained is constantly shared with global partners. The experiences were showcased during the above-mentioned event held at National Science Foundation Sri Lanka.

The IRD and its global partners were keen in sharing its CEI experiences from the inception. A poster presentation was included at the Keele University, UK’s annual Research User Group event in November 2019. That presentation focused on the success of the introduction of CEI in research which was relatively unknown in Sri Lanka prior to 2018.

This project strongly aligns with the MRC’s aims to build capacity in its researchers and improve trust across research and society. Building capacity and skills within the research team at our partner institution the IRD has been an important outcome of this project. It has facilitated continued engagement activities using social media platforms outside of grant funding, building trust and breaking down barriers between researchers and society. These skills and strategies will be applied to other research projects conducted at the IRD, amplifying its impact.

These collaborations reinforced CEI as a bidirectional knowledge mobilisation process, with Sri Lankan adaptations—such as trilingual engagement strategies and creative dissemination—informing global CEI discussions, rather than functioning solely as recipients of externally developed models.

## Discussion

This case study demonstrates that embedding CEI within LMIC research systems is less the result of isolated engagement activities and more the outcome of sustained alignment between institutional leadership, ethical infrastructure, and culturally responsive practices.

Over a seven-year period (2018–2025), CEI in Sri Lanka moved from a novel concept to an embedded practice and a recognised component of research design, governance, and dissemination.

### CEI as institutional infrastructure

A distinguishing feature of the Sri Lankan experience was the institutionalisation of CEI through ethics governance, coordination structures, and training. Integrating CEI into ERC processes provided structural legitimacy and reduced reliance on individual champions. CRIGs further supported continuity by creating formal spaces for community involvement across research cycles and studies, directly influenced study protocols, such as ensuring culturally appropriate and gender-sensitive data collection methods. This contrasts with contexts where CEI remains project-specific and vulnerable to funding cycles. integration of CEI into research training and postgraduate education, and the creation of innovative dissemination outputs such as trilingual publications, and national media coverage.

Illustrative examples from this case study suggest CEI contributed across multiple levels:

- *On research processes*: CRIGs and Ethics committee workshops supported integration of CEI into review processes.
- *On people and communities*: CEI appears to have increased awareness of research relevance, empowered community members to take part in agenda-setting, and gave traditionally marginalised groups (e.g., rural communities, twins, caregivers) a voice in shaping studies.
- *On researchers and institutions*: Exposure to CEI broadened the perspectives of Sri Lankan researchers, embedding a more participatory approach into institutional culture. Collaborations with international partners created opportunities for bidirectional learning and adaptation of frameworks.
- *On wider society*: National media engagement has normalised CEI in public discourse, while trilingual dissemination materials increased accessibility.

### Contextual and cultural adaptation of CEI

The role of context is central to CEI, as engagement practices are shaped by social, cultural, institutional, and political environments. What constitutes meaningful involvement varies across settings, and models developed in high-income countries are not always directly transferable to LMICs without adaptation. Factors such as language diversity, cultural norms, resource constraints, and existing community structures influence both the feasibility and impact of CEI. This highlights the need for context-sensitive and flexible approaches that prioritise local relevance, as emphasised in the broader CEI and participatory research literature [26, 27]. 

The context both facilitated and constrained CEI. International partnerships with Keele University and NIHR provided methodological guidance and credibility. The IRD’s longstanding ethos of working “with the people” gave CEI cultural legitimacy. However, economic and political instability, together with the COVID-19 pandemic, limited resources for face-to-face engagement. Despite these challenges, the rapid uptake of online platforms and the commitment of volunteers enabled CEI activities to continue.

### Knowledge mobilisation as the central organising logic

Knowledge mobilisation provided the connective tissue linking CEI across awareness, design, implementation, and dissemination stages. Bidirectional exchange with international partners enriched local practice, while Sri Lankan innovations—such as creative dissemination and multilingual engagement—contributed to global CEI learning.

Several process factors have proved critical to the successful integration of CEI in Sri Lankan research:

- Trilingual communication strategies (Sinhala, Tamil, English) allowed broader inclusion.
- Media partnerships amplified dissemination at no additional cost.
- Embedding CEI into ethical review processes created structural reinforcement.
- Voluntary networks and CRIGs ensured sustainability despite limited funding.
- Challenges included reliance on motivated individuals, uneven understanding of CEI across institutions, and difficulties maintaining engagement once initial project funding ended.

### IRD’s role in strategic advocacy and sustainability

Strategic advocacy played a key role in embedding CEI within policy-relevant and funding-sensitive institutions. The IRD in Sri Lanka has been engaged in community-focused initiatives since its inception in 1997, long before the concepts were formally labelled as Community Engagement and Involvement (CEI) or Patient and Public Involvement and Engagement (PPIE). CEI was not prevalent throughout Sri Lankan research and although the terminology was not in use at the time, the active participation of communities has always been a cornerstone of the IRD’s ethos. This foundational principle evolved into a structured and strategic approach, informed by international best practice but rooted in local experience.

The introduction of CEI in Sri Lanka was significantly strengthened through collaboration with Keele University in the UK, a global leader in PPIE. Keele’s decade of expertise ensured that the Sri Lankan CEI model was not only evidence-based but also co-produced with local stakeholders. Contributions included facilitating workshops, guiding discussions on integrating lived experience into research, and sharing co-production and knowledge mobilisation strategies that had proven effective in the UK [28].

Between 2018 and 2025, the IRD played a leading role in the evolution of CEI along a continuum from consultation (seeking views) to collaboration (ongoing partnership) and co-production (shared decision-making and power between researchers and communities). The examples presented in the case study demonstrate that community partners were not merely consulted but actively shaped decision-making.

### The future of CEI in Sri Lanka: theory development and implementation

This case study contributes to conceptual development by demonstrating how CEI can be adapted from PPIE frameworks developed in high-income countries to a lower-middle-income context using contextually and culturally appropriate approaches. The Sri Lankan experience highlights CEI as a form of knowledge mobilisation—a bidirectional process where international models were adapted locally, while Sri Lankan innovations (e.g., trilingual dissemination, cultural events, docudramas) contributed to the global CEI field. It also underscores the importance of co-production in building trust and embedding CEI in national research culture.

This initiative represents one of the more sustained, documented efforts in a lower-middle-income country (LMIC) to bring together diverse stakeholders and establish momentum for CEI through knowledge mobilisation at a national, society, and practitioner level, gradually normalising its role in research. Over the seven-year period from 2018 to 2025, the breadth of dissemination, multi-level networking, and engagement with both top-down and bottom-up approaches suggests CEI has influenced research culture and engagement practices in Sri Lanka. A multi-pronged strategy, reinforced by continuous media involvement, has laid a foundation to support appropriate levels of CEI in Sri Lankan research.

A crucial takeaway from this experience is the role of knowledge mobilisation in ensuring that CEI principles are not only implemented but continuously refined through shared learning. Knowledge mobilisation goes beyond dissemination by actively connecting research evidence with practice and engaging stakeholders across all levels. This conceptualisation aligns with NIHR’s definition of knowledge mobilisation as a dynamic and collaborative process that ‘paves the way to impact’ by involving knowledge users throughout the research cycle [7]. In this context, the exchange of knowledge between Sri Lanka and international collaborators, particularly Keele University, has been pivotal in embedding best practices while ensuring local adaptability. The process of co-developing CEI strategies exemplifies how bidirectional knowledge mobilisation can support improvements in research culture in LMICs, fostering more inclusive and contextually relevant engagement frameworks [29].

As CEI continues to evolve in Sri Lanka, knowledge mobilisation will remain a critical strategy for sustaining momentum, adapting to emerging challenges, and ensuring that engagement remains meaningful and impactful. However, current CEI activity often relies on the personal commitment of individual researchers and academics. Those who have embraced CEI have demonstrated its value by successfully involving appropriate partners and achieving meaningful outcomes, yet a significant challenge remains in engaging researchers who are either unaware of CEI or who do not view it as a priority. Addressing this gap will require both capacity building and a cultural shift to recognise CEI as integral rather than optional. Encouragingly, a growing awareness of CEI, fostered by IRD initiatives and collaborative efforts, may help overcome these attitudinal and institutional barriers.

IRD’s initiative to incorporate CEI into ethical reviews and empower ERCs through toolkits has gained momentum, helping CEI to be viewed as a “friend” of research rather than a barrier. The adaptation of principles from the UK’s *Ethics of Public Involvement in Research Design* [30] could be an appropriate next step, with IRD well positioned through its networks and leadership to act as a national hub for CEI.

The key challenge is to sustain this momentum beyond the first few years of introducing CEI in Sri Lanka. The current economic climate and debt restructuring create pressures that require innovative, strategic approaches to ensure CEI retains its position within the country’s research policy framework. Opportunities also exist in the education sector: the draft National Education Policy Framework 2023–2033 (National Education Policy Framework Sri Lanka, 2023) proposes major reforms, and CEI should find a niche within this evolving landscape.

With Sri Lanka’s experience, an overarching challenge for LMICs remains embedding CEI as a core element of national research culture while ensuring equitable opportunities for participation. CEI must address underlying structural inequalities and power imbalances, aligning with NIHR’s commitment to “leave no one behind” in global health research, ensuring that marginalised voices are not only included but meaningfully involved at every stage of the research cycle [31].

Finally, lobbying (strategic advocacy efforts by IRD and its partners to influence institutional and policy-level recognition of CEI) remains a key tool for placing CEI at the centre of research institutions that continually seek external funding. There is also a clear opportunity for future research in LMIC settings to evaluate which CEI approaches are most effective, both in terms of their impact on research processes and their value to the communities involved.

### Evaluation and reflection

Formal qualitative or quantitative methods to capture the impact of CEI were not applied. The findings should therefore be interpreted as practice-based insights and illustrative examples. However, narrative evidence presented in the case study highlights a range of real-life impacts of CEI. These examples help to illustrate the contribution of CEI, though systematic evaluation remains an area for future development.

## Conclusion

This narrative case study illustrates how CEI can be successfully introduced and embedded in LMIC research systems through contextually adapted, participatory approaches and sustained knowledge mobilisation. The Sri Lankan experience provides a practical model for integrating CEI into research culture, while highlighting the importance of collaborations, institutional support, policy alignment, and long-term capacity building. This iterative process reflects CEI as a bidirectional partnership: international expertise provided structure and frameworks, while Sri Lankan stakeholders ensured contextual adaptation and sustainability. Future efforts should focus on evaluating CEI impact and strengthening systems to ensure equitable and sustained participation.

## Supplementary Information

Below is the link to the electronic supplementary material.

Supplementary Material 1

## Data Availability

Not applicable.

## References

[1] Tran TA. Community engagement and involvement: enhancing research impact. OUCRU [Internet]. 2024 Jul 19 [cited 2025 Oct 3]. Available from: https://www.oucru.org/community-engagement-and-involvement-enhancing-research-impact/

[2] Community engagement and involvement | NIHR [Internet]. [cited 2025 Oct 3]. Available from: https://www.nihr.ac.uk/research-funding/global-health/community-engagement-and-involvement

[3] Community Engagement and Involvement glossary | NIHR [Internet]. [cited 2025 Oct 3]. Available from: https://www.nihr.ac.uk/research-funding/global-health/community-engagement-and-involvement/cei-glossary

[4] Epistemic justice and the knowledge commons for lifelong and lifewide [Internet]. [cited 2026 Apr 7]. Available from: https://www.unesco.org/en/articles/epistemic-justice-and-knowledge-commons-lifelong-and-lifewide-learning

[5] Brett J, Staniszewska S, Mockford C, Herron-Marx S, Hughes J, Tysall C, et al. Mapping the impact of patient and public involvement on health and social care research: a systematic review. Health Expect. 2014;17(5):637–50. 10.1111/j.1369-7625.2012.00795.x. 22809132 10.1111/j.1369-7625.2012.00795.x PMC5060910

[6] Hewlett S, Wit MD, Richards P, Quest E, Hughes R, Heiberg T, et al. Patients and professionals as research partners: Challenges, practicalities, and benefits. Arthritis Care Res. 2006;55(4):676–80. 10.1002/art.22091. 10.1002/art.22091 16874772

[7] Plan knowledge mobilisation | NIHR [Internet]. [cited 2025 Oct 9]. Available from: https://www.nihr.ac.uk/research-funding/application-support/plan-knowledge-mobilisation

[8] Anderson AM, Brading L, Swaithes L, Evans N, Fedorowicz SE, Murinas D, et al. Building trust and inclusion with under-served groups: a public involvement project employing a knowledge mobilisation approach. Res Involv Engagem. 2024;10(1):122. 10.1186/s40900-024-00647-2. 39529198 10.1186/s40900-024-00647-2 PMC11555807

[9] Wright MT, Springett J, Kongats K. What is participatory health research? In: Wright MT, Kongats K, editors. Participatory health research: voices from around the world [Internet]. Cham: Springer International Publishing; 2018 [cited 2026 Apr 9]. pp. 3–15. Available from: 10.1007/978-3-319-92177-8_1.

[10] Jinks C, Carter P, Rhodes C, Taylor R, Beech R, Dziedzic K, et al. Patient and public involvement in primary care research - an example of ensuring its sustainability. Res Involv Engagem. 2016;2(1):1. 10.1186/s40900-016-0015-1. 29062502 10.1186/s40900-016-0015-1 PMC5611572

[11] Faluyi D, Ovseiko PV, Dziedzic K, Scott F, Tulloch A, Barker C, et al. NIHR Race Equality Framework: development of a tool for addressing racial equality in public involvement. Res Involv Engagem. 2024;10(1):44. 10.1186/s40900-024-00569-z. 38715152 10.1186/s40900-024-00569-z PMC11077722

[12] Brett J, Staniszewska S, Mockford C, Seers K, Herron-Marx S, Bayliss H. The PIRICOM study: a systematic review of the conceptualisation, measurement, impact and outcomes of patients and public involvement in health and social care research [Report] [Internet]. Coventry, UK: University of Warwick; 2010 [cited 2025 Oct 3]. Available from: http://www.ukcrc.org/systematic-review-on-ppi-in-clinical-research/

[13] Lang I, King A, Jenkins G, Boddy K, Khan Z, Liabo K. How common is patient and public involvement (PPI)? Cross-sectional analysis of frequency of PPI reporting in health research papers and associations with methods, funding sources and other factors. BMJ Open. 2022;12(5):e063356. 10.1136/bmjopen-2022-063356. PubMed PMID: 35613748. 35613748 10.1136/bmjopen-2022-063356 PMC9131100

[14] Guiding Principles for CEI | NIHR [Internet]. [cited 2025 Oct 3]. Available from: https://www.nihr.ac.uk/research-funding/global-health/community-engagement-and-involvement/guiding-principles

[15] Paparini S, Green J, Papoutsi C, Murdoch J, Petticrew M, Greenhalgh T, et al. Case study research for better evaluations of complex interventions: rationale and challenges. BMC Med. 2020;18(1):301. 10.1186/s12916-020-01777-6. 33167974 10.1186/s12916-020-01777-6 PMC7652677

[16] ENHANCE Sri Lanka. – Institute for Research & Development – in Health & Social Care [Internet]. [cited 2025 Nov 19]. Available from: https://ird.lk/wpll-project/enhance-sri-lanka/

[17] Colombo Twin and Singleton Study. (CoTASS 1) – Sri Lankan Twin Registry [Internet]. [cited 2026 Apr 21]. Available from: https://sltr.ird.lk/project/colombo-twin-and-singleton-study/

[18] South Asian Early Development & Research Capacity Building Project –. Sri Lankan Twin Registry [Internet]. [cited 2026 Apr 21]. Available from: https://sltr.ird.lk/project/south-asian-early-development-research-capacity-building-project/

[19] ECLIPSE [Internet]. [cited 2025 Oct 3]. Available from: https://eclipse-community.com/

[20] GAVESHANA 36 – Gaveshana [Internet]. [cited 2025 Oct 9]. Available from: https://gaveshana.lk/magazine/gaveshana-36/

[21] Colombo Twin and Singleton Study – 3 – Pilot Study 2 – Institute for Research & Development – in Health & Social Care [Internet]. [cited 2026 Apr 21]. Available from: https://ird.lk/wpll-project/test-project/

[22] Jayasinghe OS, Hewamalage A, Sumathipala A, Sikander S, Rahman A. Nothing about us, without us: Translation and cultural adaptation of the Thinking Healthy Programme for perinatal depression to Sri Lankan context using a co-designing approach. Asian J Psychiatry. 2025;110:104564. 10.1016/j.ajp.2025.104564. 10.1016/j.ajp.2025.104564 40482290

[23] Surenthirakumaran DR, Kumaran DS, Shribavan DK, Shanmuganathan Y. Community engagement and involvement in research: a local experiences.

[24] Publications – ECLIPSE [Internet]. [cited 2025 Oct 9]. Available from: https://eclipse-community.com/publications/

[25] Thinking Healthy [Internet]. [cited 2025 Oct 9]. Available from: https://www.who.int/publications/i/item/WHO-MSD-MER-15.1

[26] Lammons W, Buffardi AL, Marks D. Measuring impacts of patient and public involvement and engagement (PPIE): a narrative review synthesis of review evidence. Res Involv Engagem. 2025;11(1):76. 10.1186/s40900-025-00748-6. 40616180 10.1186/s40900-025-00748-6 PMC12228192

[27] Ahmed M, McLean J, Donaldson C, Roy MJ, Baker R. Creating the conditions for meaningful and effective PPIE in community-based public health research: learning from a UK-wide lived experience panel. Res Involv Engagem. 2025;11(1):85. 10.1186/s40900-025-00727-x. 40676654 10.1186/s40900-025-00727-x PMC12273268

[28] Anderson AM, Brading L, Swaithes L, Evans N, Fedorowicz SE, Murinas D, et al. Building trust and inclusion with under-served groups: a public involvement project employing a knowledge mobilisation approach. Res Involv Engagem. 2024;10(1):122. 10.1186/s40900-024-00647-2. 39529198 10.1186/s40900-024-00647-2 PMC11555807

[29] Herath T, Guruge D, Fernando M, Jayarathna S, Senarathna L. The effect of a community-based health promotion intervention to change gender norms among women in a rural community in Sri Lanka. BMC Public Health. 2018;18(1):977. 10.1186/s12889-018-5914-7. 30081873 10.1186/s12889-018-5914-7 PMC6080371

[30] Staley K, Elliott J. Public involvement could usefully inform ethical review, but rarely does: what are the implications? Res Involv Engagem. 2017;3(1):30. 10.1186/s40900-017-0080-0. 29238613 10.1186/s40900-017-0080-0 PMC5724337

[31] What. does it mean to take a “leave no one behind“ approach to community engagement and involvement in global health research? | NIHR [Internet]. [cited 2025 Oct 9]. Available from: https://www.nihr.ac.uk/what-does-it-mean-take-leave-no-one-behind-approach-community-engagement-and-involvement-global-health-research

