# Supplementary material for: Community engagement and involvement in Sri Lanka: a knowledge mobilisation case study
Source: Res Involv Engagem. 2026 Jun 16;12:93. doi: 10.1186/s40900-026-00923-3 (PMC13270724; doi:10.1186/s40900-026-00923-3)
Supplement: Supplementary file 1 — Supplementary Material 1 [file 40900_2026_923_MOESM1_ESM.docx]

# Completed GRIPP2 Checklist with Page References

| Section | Checklist Item | Reported on Page(s) |
| --- | --- | --- |
| Section 1: Abstract of paper | | |
| 1a: Aim | Report the aim of the study | Page 1 |
| 1b: Methods | Describe the methods used by which patients and the public were involved | Page 1 |
| 1c: Results | Report the impacts and outcomes of CEI in the study | Page 1 |
| 1d: Conclusions | Summarise the main conclusions of the study | Page 2 |
| 1e: Keywords | Include CEI, patient and public involvement, or alternative terms as keywords | Page 3 |
| Section 2: Background to paper | | |
| 2a: Definition | Report the definition of CEI used in the study and how it links to comparable studies | Page 3 |
| 2b: Theoretical underpinnings | Report the theoretical rationale and any theoretical influences relating to CEI in the study | Page 4 |
| 2c: Concepts and theory development | Report any conceptual or theoretical models, or influences, used in the study | Pages 4-5 |
| Section 3: Aims of paper | Report the aim of the study | Page 6 |
| Section 4: Methods of paper | | |
| 4a: Design | Provide a clear description of methods by which patients and the public were involved | Pages 6-8 |
| 4b: People involved | Provide a description of patients, carers, and the public involved with the CEI activity in the study | Pages 8-9 |
| 4c: Stages of involvement | Report on how CEI is used at different stages of the study | Pages 9-11 |
| 4d: Level or nature of involvement | Report the level or nature of CEI used at various stages of the study | Pages 11-12 |
| Section 5: Capture or measurement of PPI impact | | |
| 5a: Qualitative evidence of impact | If applicable, report the methods used to qualitatively explore the impact of CEI in the study | Page 12 |
| 5b: Quantitative evidence of impact | If applicable, report the methods used to quantitatively measure or assess the impact of CEI | N/A |
| 5c: Robustness of measure | If applicable, report the rigour of the method used to capture or measure the impact of CEI | N/A |
| 6: Economic assessment | If applicable, report the method used for an economic assessment of CEI | N/A |
| Section 7: Study results | | |
| 7a: Outcomes of PPI | Report the results of CEI in the study, including both positive and negative outcomes | Pages 12–22 |
| 7b: Impacts of PPI | Report the positive and negative impacts that CEI has had on the research, individuals, and wider impacts | Pages 22-23 |
| 7c: Context of PPI | Report the influence of any contextual factors that enabled or hindered the process or impact of CEI | Pages 22-23 |
| 7d: Process of PPI | Report the influence of any process factors that enabled or hindered the impact of CEI | Pages 23-24 |
| 7ei: Theory development | Report any conceptual or theoretical development in CEI that have emerged | Pages 24 |
| 7eii: Theory development | Report evaluation of theoretical models, if any | N/A |
| 7f: Measurement | If applicable, report all aspects of instrument development and testing | N/A |
| 7g: Economic assessment | Report any information on the costs or benefit of CEI | N/A |
| Section 8: Discussion and conclusions | | |
| 8a: Outcomes | Comment on how CEI influenced the study overall. Describe positive and negative effects | Pages 22 |
| 8b: Impacts | Comment on the different impacts of CEI identified in this study and how they contribute to new knowledge | Pages 22-23 |
| 8c: Definition | Comment on the definition of CEI used and whether or not you would suggest any changes | N/A |
| 8d: Theoretical underpinnings | Comment on any way your study adds to the theoretical development of CEI | Pages 23-24 |
| 8e: Context | Comment on how context factors influenced CEI in the study | Page 24-26 |
| 8f: Process | Comment on how process factors influenced CEI in the study | Page 24-26 |
| 8g: Measurement and capture of PPI impact | If applicable, comment on how well CEI impact was evaluated or measured in the study | N/A |
| 8h: Economic assessment | If applicable, discuss any aspects of the economic cost or benefit of CEI | N/A |
| 8i: Reflections/critical perspective | Comment critically on the study, reflecting on the things that went well and those that did not | Pages 25-27 |
